# Supplementary figures and images for: SlugAtlas, a histological and 3D online resource of the land slugs Deroceras laeve and Ambigolimax valentianus
Source: PLoS One. 2024 Oct 22;19(10):e0312407. doi: 10.1371/journal.pone.0312407 (PMC11495586; doi:10.1371/journal.pone.0312407)

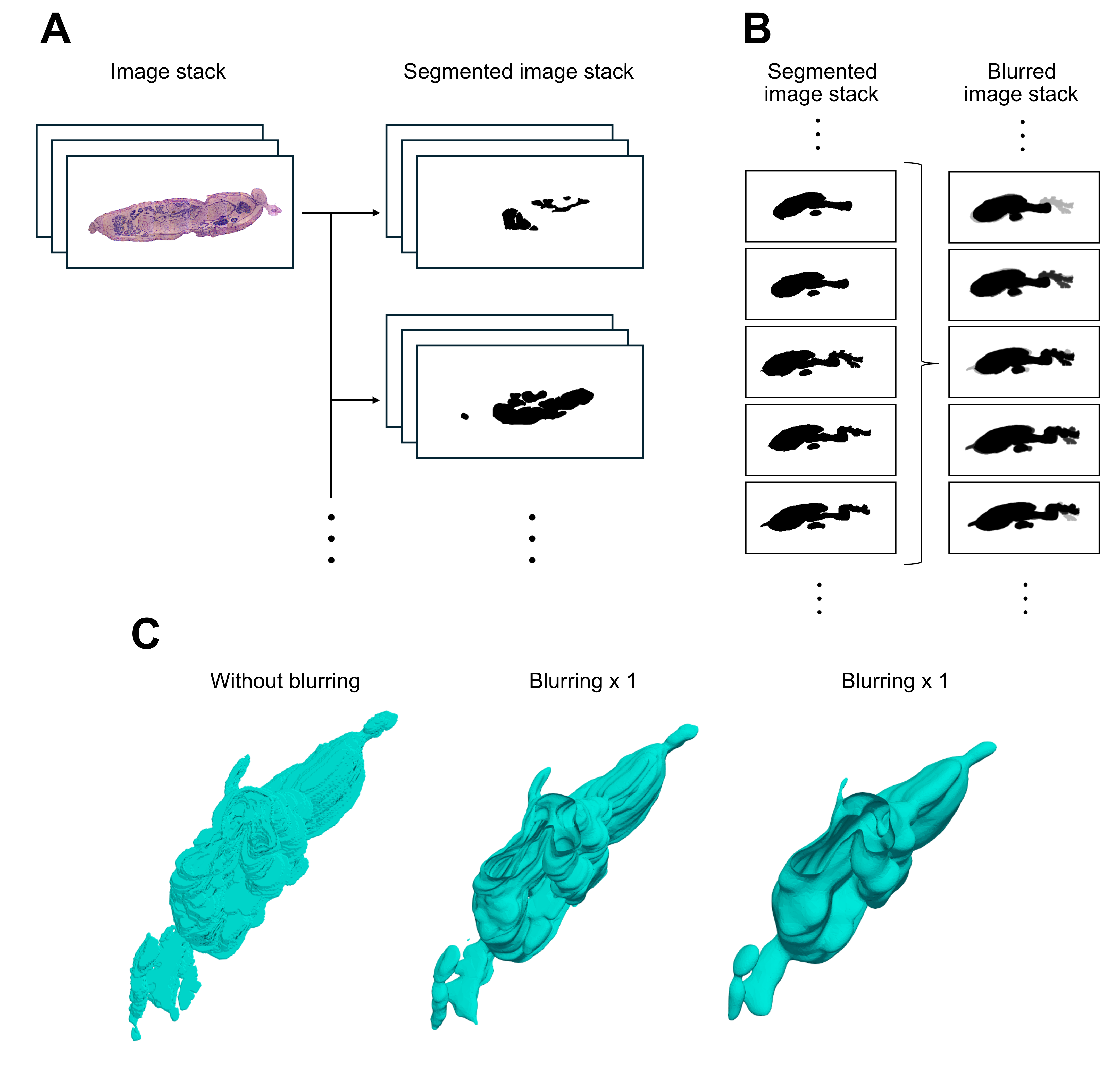

Supplement: S1 Fig — (A) Example of segmentation. (B) Example of 3D blurring of the digestive tract. (C) Comparison between 3D models of the digestive tract without blurring and with blurring. (TIF) [file pone.0312407.s001.tif]

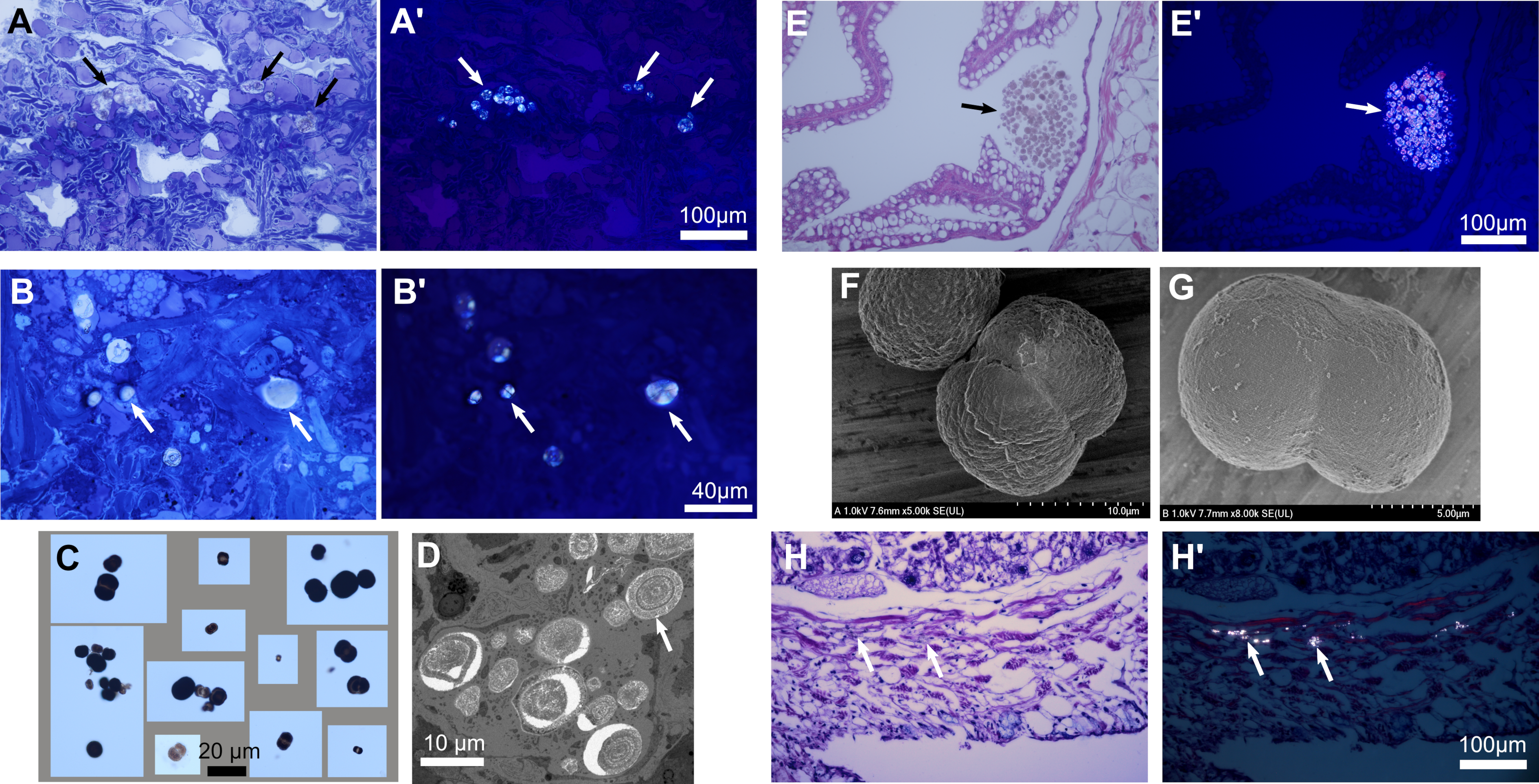

Supplement: S2 Fig — (A-G) Concretions observed in D. laeve and (H and H’) in A. valentianus. (A and B) Bright-field micrographs of semithin sections of the body wall and (A’ and B’) the same fields with DIC optics showing refringent concretions (arrows). (C) Concretions extracted from the body wall that were heat-treated (concretions from different micrographs were combined in a single image and are shown at the same scale). (D) Transmission electron micrograph of concretions showing concentric rings in cross-section. (E and E’) Histological section of the kidney showing concretions in the renal lumen in bright-field and DIC optics of the same field, respectively (arrows). (F) Scanning electron micrograph of a concretion extracted from the body wall. (G) Concretion extracted from the kidney. (H and H’) Concretions observed in the subepidermal connective in bright-field and DIC optics of the same field in A. valentianus (arrows). (TIF) [file pone.0312407.s002.tif]

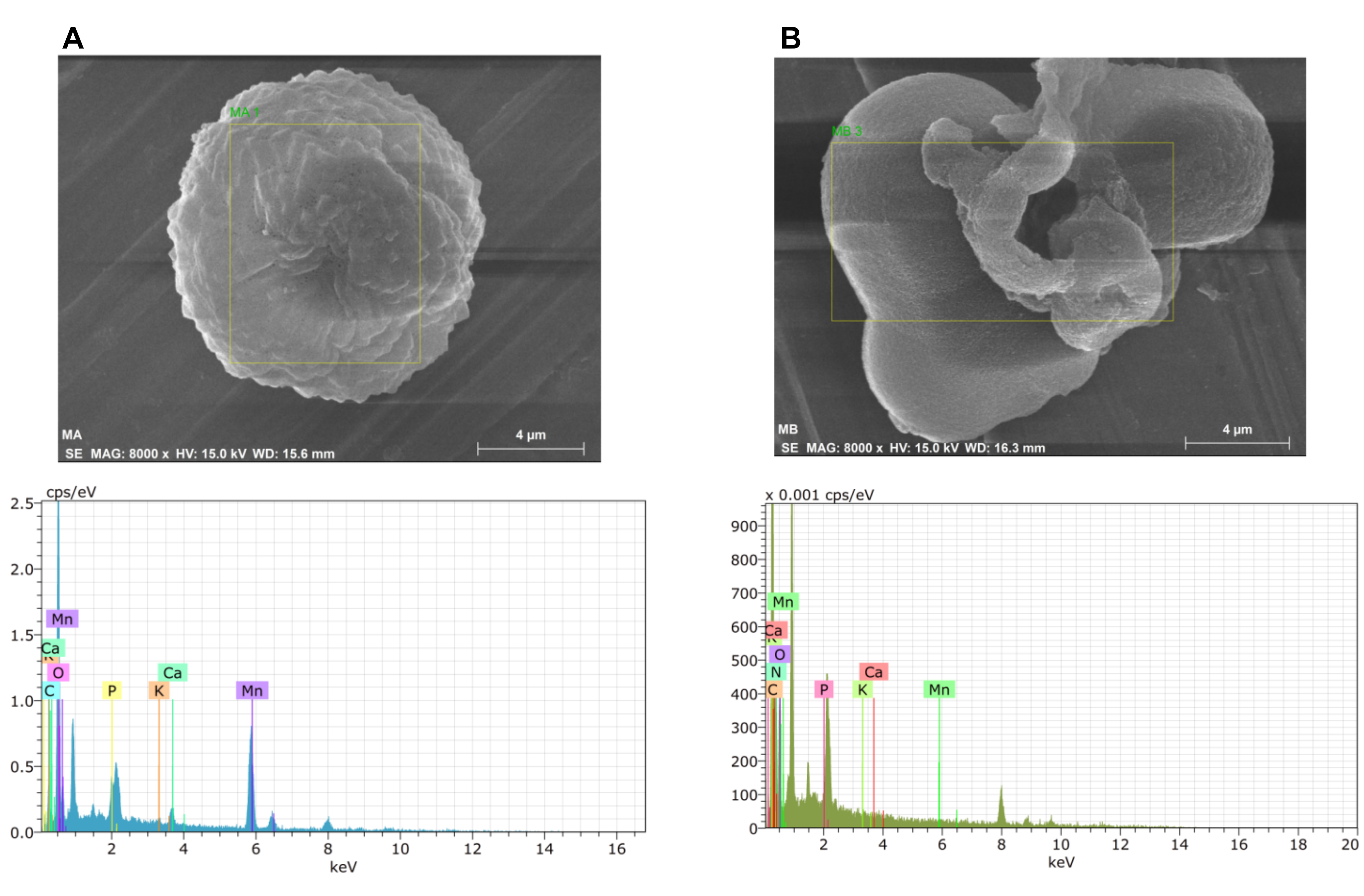

Supplement: S3 Fig — (A) Concretions obtained from the body wall. (B) Concretions obtained from the kidney. Top micrographs on both, scanning electron microscopy images; bottom on both, EDS plot. (TIF) [file pone.0312407.s003.tif]
