# Supplementary material for: SlugAtlas, a histological and 3D online resource of the land slugs Deroceras laeve and Ambigolimax valentianus
Source: PLoS One. 2024 Oct 22;19(10):e0312407. doi: 10.1371/journal.pone.0312407 (PMC11495586; doi:10.1371/journal.pone.0312407)
Supplement: S1 Table — (DOCX) [file pone.0312407.s004.docx]

**Table S1.** Sequences of the mitochondrial gene for cytochrome oxidase subunit 1 used for multiple alignment.

| **Genus** | **Species** | **GeneBank Accession** |
| --- | --- | --- |
| *Ambigolimax* | *valentianus* | PP854456 (this work) |
|  |  | JX435832.1 |
|  |  | KF894276.1 |
|  |  | KF894281.1 |
|  |  | KF894290.1 |
|  |  | AM259710.1 |
|  |  | AM259711.1 |
|  |  | MG747674.1 |
|  |  | MG747676.1 |
|  |  | MG747678.1 |
|  |  | MG747681.1 |
|  |  | MG747684.1 |
|  |  | MG747685.1 |
|  |  | MG747686.1 |
|  |  | MG799141.1 |
|  |  | MG799142.1 |
|  |  | MG799143.1 |
|  |  | MG799144.1 |
|  |  | MG799145.1 |
|  | *marginata* | FJ606455.1 |
|  |  | KF894368.1 |
|  |  | KF894372.1 |
|  |  | KF894377.1 |
|  | *nyctelius* | KF894237.1 |
|  |  | KF894238.1 |
|  |  | KF894251.1 |
|  |  | KF894253.1 |
|  |  | KF894254.1 |
|  |  | KF894302.1 |
|  |  | KF894303.1 |
|  |  | MG747671.1 |
|  |  | MG747672.1 |
|  |  | MG747673.1 |
|  |  | MG747675.1 |
|  |  | MG747677.1 |
|  |  | MG747679.1 |
|  |  | MG747680.1 |
|  |  | MG747682.1 |
|  |  | MG747683.1 |
|  |  | MG799133.1 |
|  |  | MG799134.1 |
|  |  | MG799135.1 |
|  |  | MG799136.1 |
|  |  | MG799137.1 |
|  |  | MG799138.1 |
|  |  | MG799139.1 |
|  |  | MG799140.1 |
|  |  | MG856342.1 |
| *Deroceras* | *laeve* | PP854454 (this work) |
|  |  | PP854455 (this work) |
|  |  | KM611814.1 |
|  |  | KM611853.1 |
|  |  | KM612156.1 |
|  |  | KM611889.1 |
|  |  | KM611903.1 |
|  |  | KM611984.1 |
|  |  | KM612156.1 |
|  |  | KM612188.1 |
|  |  | KM612199.1 |
|  |  | KX959491.1 |
|  |  | KX959492.1 |
|  |  | KX959493.1 |
|  |  | KX959494.1 |
|  |  | KX959495.1 |
|  |  | KX959496.1 |
|  |  | KX959497.1 |
|  |  | KX959498.1 |
|  |  | KX959499.1 |
|  |  | KX959500.1 |
|  |  | KX959501.1 |
|  |  | EF128217.1 |
|  |  | HM584699.1 |
|  |  | JX435885.1 |
|  |  | KF894260.1 |
|  |  | KF894261.1 |
|  |  | KF894262.1 |
|  |  | KF894277.1 |
|  |  | KF894311.1 |
|  |  | KF894344.1 |
|  |  | KF894364.1 |
|  | *reticulatum* | KT705645.1 |
|  |  | KT705739.1 |
|  |  | KM611812.1 |
|  |  | KM611817.1 |
|  |  | KM612024.1 |
|  |  | KM612116.1 |
|  |  | FJ481179.1 |
|  |  | KX959502.1 |
|  |  | KX959503.1 |
|  |  | KX959504.1 |
|  |  | KF894235.1 |
|  |  | KF894308.1 |
|  |  | KF894313.1 |
|  |  | KF894363.1 |
|  |  | KF894376.1 |
|  |  | KF894378.1 |
|  | *invadens* | KF894269.1 |
|  |  | KF894244.1 |
|  |  | KF894245.1 |
|  |  | KF894259.1 |
|  |  | KF894343.1 |
|  |  | KF894370.1 |
|  |  | KX959488.1 |
|  |  | KX959489.1 |
|  |  | KX959490.1 |
|  |  | JQ743070.1 |
|  |  | JN248295.2 |
|  |  | JN248296.2 |
|  |  | JN248301.1 |
|  |  | JN248302.2 |
|  |  | JN248315.2 |
|  | *golcheri* | JN248291.1 |
|  |  | JN248292.1 |
|  |  | JN248293.1 |
|  | *panormitanum* | JN248310.1 |
|  |  | JN248312.1 |
|  |  | KM611878.1 |
|  |  | KM612026.1 |
|  |  | KM612148.1 |
|  |  | KM612186.1 |
|  |  | KF894327.1 |
|  | *agreste* | KF894247.1 |
|  |  | KF894375.1 |
| *Limax* | *flavus* | FJ481181.1 |
|  |  | KF894304.1 |
|  |  | KF894331.1 |
|  | *cinereoniger* | FJ606464.1 |
|  |  | FJ606465.1 |
|  | *maximus* | JN248294.2 |
|  |  | KM612139 |
| *Bielzia* | *coerulans* | JX435825.1 |
|  |  | JX435829.1 |
| *Malacolimax* | *tenellus* | JX435836.1 |
|  |  | KF894297.1 |
|  |  | KF894349.1 |
|  |  | KF894369.1 |
|  |  | KF894379.1 |
