# Supplementary material for: SlugAtlas, a histological and 3D online resource of the land slugs Deroceras laeve and Ambigolimax valentianus
Source: PLoS One. 2024 Oct 22;19(10):e0312407. doi: 10.1371/journal.pone.0312407 (PMC11495586; doi:10.1371/journal.pone.0312407)
Supplement: S2 Table — (DOCX) [file pone.0312407.s005.docx]

**Supplementary Table 2.** Elemental composition of concretions.

|  | **Normalized mass (%)** | |
| --- | --- | --- |
| **Element** | **Body wall** | **kidney** |
| C | 12.17 | 36.09 |
| N | 0 | 37.72 |
| O | 44.47 | 25.07 |
| P | 1.76 | 1.08 |
| K | 0.64 | 0.06 |
| Ca | 1.96 | 0.04 |
| Mn | 39.02 | 0 |

Average of two EDS determinations is shown.
