# Supplementary material for: SlugAtlas, a histological and 3D online resource of the land slugs Deroceras laeve and Ambigolimax valentianus
Source: PLoS One. 2024 Oct 22;19(10):e0312407. doi: 10.1371/journal.pone.0312407 (PMC11495586; doi:10.1371/journal.pone.0312407)
Supplement: Attachment — Submitted filename: Response_to_reviewers.docx [file pone.0312407.s008.docx]

PONE-D-24-25268

SlugAtlas, a histological and 3D online resource of the land slugs Deroceras laeve and Ambigolimax valentianus

PLOS ONE

Editor’s comments:

COMMENT: To strengthen your paper, I recommend considering a broader context for your work. Specifically, you might consider including comparisons with other gastropod molluscs, particularly the well-studied Aplysia. This could provide a richer framework for your findings and enhance their significance within the field.

RESPONSE: Additional information has been included in the Introduction referencing examples of other gastropod models used for different research purposes. Moreover, throughout Discussion, results of this study are now contrasted with other sea and land gastropods including Aplysia and with some bivalves as well. Also, the morphological aspects of the regeneration of the tail, are compared with those of vertebrates and other invertebrates including planarias, the snail Pomacea canaliculata, the cephalopod Octopus, and Aplysia.

COMMENT: If you would like to make changes to your financial disclosure, please include your updated statement in your cover letter.
RESPONSE: An updated version of the financial disclosure with funding information has been included at the end of the cover letter.

COMMENT: Guidelines for resubmitting your figure files are available below the reviewer comments at the end of this letter.
RESPONSE: All main figures have been edited to comply with the journal ́s guidelines, uploaded to the Preflight Analysis and Conversion Engine (PACE) digital diagnostic tool, and resubmitted.

COMMENT: If applicable, we recommend that you deposit your laboratory protocols in protocols.io to enhance the reproducibility of your results.
RESPONSE: We plan to submit at a later time a separate protocols article with the methods that are specific for these model animals.

Reviewers' comments:

Reviewer #1
COMMENT: In this work the authors performed the first characterization of whole adult individuals of both D. laeve and A. valentianus in three planes of section and described aspects of growth and maintenance of them. The digitalized imaging data were compiled into an online server-based anatomical atlas.
While collecting valuable anatomical data, the critical claims in justifying the significance of the dataset as paving the road towards new model organism in studying regeneration should be further justified. In the work, the authors performed dietary changes that induced degrowth and regrowth of the animal, and in sea slugs it has been recently found that fully body could have regenerated with great capacity. Thus, a body amputation and regeneration inspection (with potentially EdU staining for cell growth) of D. laeve should be conducted to fully justify that land slug is a group of organisms capable of self-regeneration. The regeneration related genes should be aligned and inspected with other self-regenerating animals to leverage bioinformatics for further getting how confident we may believe land slugs as a suitable model organisms for regeneration.
RESPONSE: In agreement with the reviewer’s suggestion of including an exploration of regeneration in this model system, we have added a description of the morphological aspects of regeneration of the tail of Deroceras laeve upon amputation as well as an inspection of cell proliferation using the marker of DNA synthesys F-ara- EdU (Figure 17). This also required the inclusion of new sections or paragraphs in Materials and Methods (Amputation and F-ara-EdU labeling, line 128), Results (Tail regeneration in Deroceras laeve, line 873), Discussion (line 1034), and Conclusions (line 1075).
We also coincide in that performing molecular analysis will allow comparative studies with other animals capable of regeneration. One current difficulty is that a transcriptome is not yet available for D. laeve. For this reason we have initiated analysis of the molecular processes involved in the regeneration of the tail entailing a de novo assembly of a transcriptome which will continue beyond the scope of the current work.

COMMENT: Currently the SlugAtlas website contains only histological data, and the phylogenetic studies performed in the work should be conveniently visualized, as genome and evolutionary information is highly desirable for molecular biology studies.

RESPONSE: We included in the SlugAtlas webpage the results of the phylogenetic analysis described in the manuscript along with links to pages with information on the stylommatophoran species employed in such analysis. Additionally, SlugAtlas will incorporate references to transcriptomic and genomic data as we make it available and this is stated now in Conclusions.

COMMENT: Potentially beyond the scope of the capacity of this revision, the capability and protocol for producing modified clones of organism with RNAi/CRSIPR is also crucial for any molecular perturbation of the animal. A de novo assembly of transcriptome and the profiling of gene expression dynamics during degrowth and regrowth will also provide more insights translatable into comparative development biology and regeneration studies in the nowadays research context.

RESPONSE: In agreement with the reviewer, although beyond the scope of this manuscript, we are currently performing a study on the cellular and gene control of degrowth and regrowth employing a transcriptomic approach. Efforts are also underway to develop gene perturbation methods that will continue in subsequent studies.

Reviewer #2:

COMMENT: This is a detailed and thorough report of histological sections through the body of two slugs in the Order Stylommatophora. The quality of the data is high, and the online Slug Atlas is a potentially valuable resource. It is simple to access this digital atlas and the organs are fairly clear. My major reservation is the limited accessibility. For example, there is no discussion of the relationship between these two pulmonate slugs and other heterobranchs.
RESPONSE: Following the reviewer's observation, we have included in the first paragraph of the Introduction the following two sentences: “The Order Stylommatophora is the most abundant group of land slugs and snails. It belongs to the Subclass Heterobranchia also encompassing marine slugs and snails” (line 42).

Moreover, throughout Discussion, results with D. laeve and A. valentianus are contrasted with those of other heterobranchs, caenogastropods, bivalves, and cephalopods.

COMMENT: According to Wikipedia, “The two strong synapomorphies of Stylommatophora are a long pedal gland placed beneath a membrane and two pairs of retractile tentacles.” We should be told this at the outset of the paper.
RESPONSE: We now state clearly that retractile tentacles and a suprapedal gland isolated from the visceral cavity are stylommatophoran synapomorphies (line 50).

COMMENT: A number of terms merit explanation early on in the MS. Degrowth, which is a simple concept, should be clearly explained.
RESPONSE: Following the reviewer's suggestion, the following terms have been defined in the text:

limacization (line 57)
degrowth /regrowth (lines 65-67) cleptoplasty (line 73)
allometry (lines 105-106)
F-actin staining with phalloidin (line 467) diaulic (line 841)
protandry (line 1030)

COMMENT: Some anatomical structures deserve introduction early in Results or in the Introduction. For example: suprapedal gland and Semper’s organ (is this a neurosecretory gland?).
RESPONSE: Following the reviewer's suggestion, the following structures have been described the first time they are mentioned in Introduction or Results:

rhinophore (line 53)
ommatophore (line 55)
pneumostome (lines 60, 439)
radula (line 273)
circumesophageal nerve ring (line 381) integument (line 407)

subepidermal connective (line 410) mantle (line 434)
free mantle (line 436)
concretions (line 446)

ovotestis (line 797)
Semper’s organ. Indeed, as stated in the manuscript (line 554), it has been proposed that this organ is neurosecretory. We speculate that its two main components may be connected in such a way that sensory inputs from its external ciliated plate may trigger exocrine or endocrine secretion via a local motor response, although central coordination by the cerebral ganglia may occur as well.

COMMENT: Line 343 “sexual dimorphism found only in A. valentianus.” This seems to be a confused description as both species are hermaphrodites. (On line 895, this is apparently clarified, “juvenile A. valentianus appeared to have mostly male features and the older individuals had hermaphroditic traits,” but this should be explained earlier.). Are the juvenile A. valentianus sexually active, or is this simply a developmental stage where they are sexually immature?

RESPONSE: We have eliminated the use of the term “Sexual dimorphism” as it is indeed confusing and in Discussion we described in better detail the differences in the sexual organs of A. valentianus in different stages of life also defining the term "protandry" (lines 1024-1032). Juveniles of Agriolimacidae have been observed to be sexually mature functioning predominantly as males producing sperm cells while older animals produce both male and female gametes (Wiktor, 2000).

COMMENT: Line 353. “sensory neurons” in Figure 6. This term is only used here, and there is no indication in the micrographs of Fig 6 as to the location of sensory neurons. This also raises the question of how neurons were identified.
RESPONSE: Reference to sensory neurons was eliminated as no specific staining method for this type of cells was used in this study.

Wording comments.

COMMENT: Line 42. “specially slugs” should perhaps be “especially slugs” or this might be clearer as “This is especially true of slugs, which lack ..”
RESPONSE: The suggested change has been made (line 45).

COMMENT: Line 53. “and associated to the air exchange pore” might read better as “adjacent to the air exchange pore”
RESPONSE: The suggested change has been made (line 60).

COMMENT: Line 72. “Atlantic Islands” are defined differently depending on the reference, so it would be helpful to list them specifically. Is this Canaries, Madeira, Azores and Cape Verde Islands
RESPONSE: Since the oldest reports of this species are from the Iberian peninsula and the reports in diverse islands are more recent and likely to be secondarily introduced, we have eliminated reference to the latter (line 81).

COMMENT: Line 102 “a saturated solution of menthol in ethanol.” I could not understand this, as these two alcohols are totally miscible in water.
RESPONSE: Menthol is a monoterpenoid which as a white crystalline solid has low solubility in water. For that reason we made a saturated solution adding an excess of menthol to absolute ethanol such that an insoluble precipitate remained after vigorous mixing. This allowed us to measure small amounts to dilute in water 1:400 as described in the manuscript. A more detailed description of the procedure is now included (lines 123-126).
