# Supplementary material for: SlugAtlas, a histological and 3D online resource of the land slugs Deroceras laeve and Ambigolimax valentianus
Source: PLoS One. 2024 Oct 22;19(10):e0312407. doi: 10.1371/journal.pone.0312407 (PMC11495586; doi:10.1371/journal.pone.0312407)
Supplement: Attachment — Submitted filename: Response_to_reviewers_R2.docx [file pone.0312407.s009.docx]

PONE-D-24-25268

SlugAtlas, a histological and 3D online resource of the land slugs Deroceras laeve and Ambigolimax valentianus

PLOS ONE

Editor’s comments:

COMMENT: Please review your reference list to ensure that it is complete and correct. If you have cited papers that have been retracted, please include the rationale for doing so in the manuscript text, or remove these references and replace them with relevant current references. Any changes to the reference list should be mentioned in the rebuttal letter that accompanies your revised manuscript. If you need to cite a retracted article, indicate the article’s retracted status in the References list and also include a citation and full reference for the retraction notice.

RESPONSE: The reference list has been confirmed to be complete and correct.

Comments of Reviewer #2

COMMENT: A very minor point is that there seems to be excessive use of abbreviations. Perhaps some of these are primarily used in the atlas, but if an abbreviation is not used at least 2-3 times in the text, there is no benefit to using the abbreviation in the MS, as opposed to the anatomical atlas. One clear example is sec, an abbreviation for subepidermal connective; this is an unnecessary abbreviation, as it may only be used a single time. For example, “only the body wall was affected including the epidermis and the sec” (line 877) is confusing.

RESPONSE: We have reduced the use of abbreviations throughout the text and only use them to label structures in the figures. The abbreviated form of “sec” is provided on its first mention in the main text in reference to Figure 6 (line 410) and in its Legend (Line 430). The spelled out form, however, is used throughout the text (lines 414, 419, 431, 443, 447, 877, 992, and 1114).

COMMENT: Line 89. “been a valuable tool … in recent works transcriptomic resources of its nervous system and for embryological studies have been developed.” These two areas – transcriptomic resources of its nervous system and “for embryological studies” – are not parallel. This sentence needs to be rewritten.

RESPONSE: The suggested change has been made (line 86).

COMMENT: Line 104. “Hence, it is possible to envision that this resource will be of use to study in gastropods, among other subjects, anatomy, stem cell biology, regeneration, embryology, and the control of body proportions or allometry.” This sentence should be rewritten, as it does not quite make sense. Perhaps it could be worded “Hence, this resource will potentially be useful in various areas of research in gastropods, including anatomy, stem cell biology, regeneration, embryology, and allometry, the control of body proportions.”

RESPONSE: The suggested change has been made (line 103).

COMMENT: Line 130. “clean cut of a razor blade, after which were transferred”. This sentence needs a subject before “were transferred,” such as “they.”

RESPONSE: The suggested change has been made (line 130).

COMMENT: Line 555. The Semper’s organ is a dual sensory-glandular structure that contains a ganglion connected with a subepidermal sensory component with a glomerular organization akin to that of the tentacular digitiform ganglia, linked to a ciliated epithelial plate facing the ventral side of the animal at the mouth lobes just anterior to the prebuccal groove.” This should be split into two or three sentences, and the wording clarified.

RESPONSE: The suggested change has been made (line 554).

COMMENT: Line 1030. “and transition from male to hermaphrodite is known as protandry” should be a separate sentence, e.g. “This transition from male to hermaphrodite is known as protandry.”

RESPONSE: The suggested change has been made (line 1028).

COMMENT: Line 1035. “We observed that the amputated face is covered by a transition epidermis” should probably be “amputation face.” This is a subtle difference, but as written it suggests that a face was surgically removed.

RESPONSE: The suggested change has been made (line 1035).
